# Unsedated cerebrovascular reactivity imaging is well-tolerated in children

**DOI:** 10.1007/s00247-025-06350-y

**Published:** 2025-08-13

**Authors:** Alyssa E. Smith, Josiah B. Lewis, Jingyi Zhang, Igor Dedkov, Heather Roberts, Madison Streb, Michael M. Binkley, Amy Mirro, Jerrel Rutlin, Barbra Giourgas, Joshua S. Shimony, Melanie E. Fields, Kristin P. Guilliams

**Affiliations:** 1 Washington University in St. Louis, 660 S Euclid Ave MSC 8111-43-1260, St Louis, MO 63110 United States; 2 Saint Louis University, St Louis, United States; 3 Medical University of South Carolina, Charleston, United States

**Keywords:** Magnetic resonance imaging, Pediatric, Cerebrovascular reactivity, Sickle cell disease, Carbon dioxide

## Abstract

**Background:**

Unsedated MRI use in pediatric clinical and research settings is often feasible, but advanced imaging techniques like cerebrovascular reactivity (CVR) may affect tolerability of unsedated MRIs. Exogenous carbon dioxide (CO_2_) provides a CVR vasodilatory challenge, but its impact on unsedated children’s MRI tolerability is unknown.

**Objective:**

We hypothesized that children would tolerate MRI with exogenous CO_2_ as well as children undergoing only MRI.

**Materials and methods:**

Children with and without sickle cell disease and/or reactive airway disease participated in prospective, single-site unsedated MRI observational studies and completed a post-scan questionnaire. A RespirAct® device delivered CO_2_ during the scan for MRI-measured CVR. Head displacement across frames quantified motion. Tolerability was defined as MRI study completion without lasting symptoms or serious adverse events.

**Results:**

One-hundred children participated, with a median age of 14.0 years [11.0, 16.3]. Sickle cell disease and/or reactive airway disease was present in 35% (35/100) and 16% (16/100), respectively. CVR sessions occurred in 75 participants, while 25 had MRI only. All children tolerated and completed the scans; 77% (58/75) had usable CVR data. Motion was similar between those with and without CVR (*P*=0.33). Children undergoing CVR were more likely to report symptoms, mainly shortness of breath (42/75 vs 3/25; *P*<0.001), compared to MRI only. Eleven children reported mild, temporary symptoms post-CVR scan; all resolved within a few hours. No serious adverse events occurred. Three children (CVR group) reported unwillingness to repeat the scan.

**Conclusion:**

Children tolerate MRI with exogenous CO_2_ for CVR measurement with mild, predictable transient symptoms.

**Graphical Abstract:**

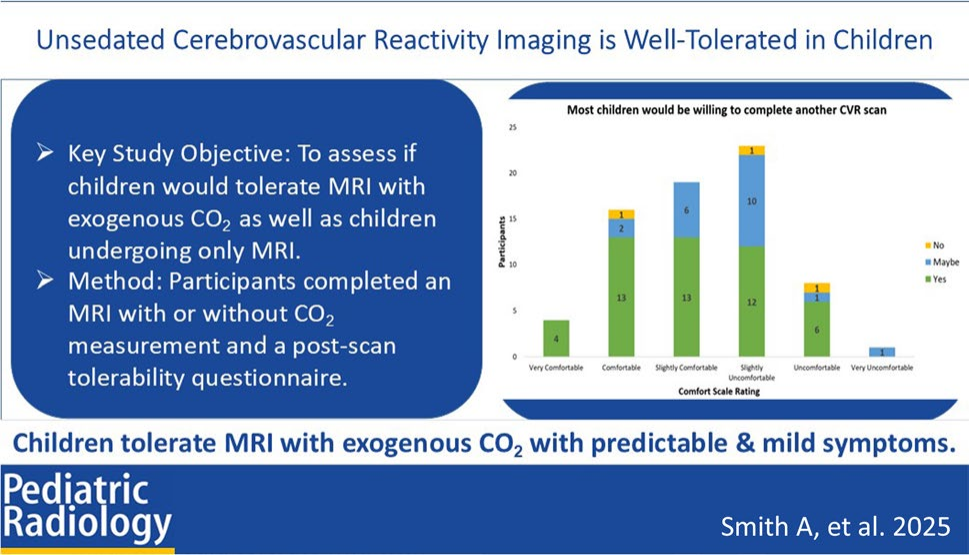

## Introduction

Advanced MRI techniques can ascertain markers of cerebral hemodynamics and cerebral blood flow (CBF), including cerebrovascular reactivity (CVR). CVR is defined as a change in CBF per change in arterial CO_2_. Regional blood flow change can be assessed by measuring the change in the blood-oxygen-level-dependent (BOLD) MRI signal [1, 2]. Arterial CO_2_, often estimated by exhaled end-tidal CO_2_, can be influenced by a stimulus that alters CO_2_ via breath holding, acetazolamide, or exogenous CO_2_. RespirAct® (4th generation, Thornhill Research, Inc Toronto, Canada) is an MR-compatible device that administers inhaled CO_2_ to the patient via a sealed mask, maintaining participants’ individual end-tidal CO_2_ and titrating CO_2_ levels accordingly. The RespirAct® delivers a computer-controlled precise targeted CO_2_ stimulus during the experiment which can be aligned with the BOLD time signal to measure the CVR [3].

CVR measurement is an increasingly valuable tool in cerebrovascular imaging research, as CVR reflects the capability of CBF to change, or autoregulate, and impaired CVR has been associated with increased risk of stroke [4]. Accurate measurement of CVR can be helpful in the study of pediatric populations at risk for vasculopathy or stroke, such as children with sickle cell disease [5]. Few studies have examined CVR in children, and it is unknown how adding another procedure of inhaled and controlled CO_2_ will impact the tolerability of MRI in children [6–8].

MRI is commonly used in pediatrics for clinical diagnostics and brain imaging research. Unlike adult patients, sedation is often given to younger aged children to maintain stillness throughout the study duration in the setting of anxiety surrounding the noise and enclosed nature of the scanner [9–12]. This can be particularly challenging for MRI data that require wakefulness and participation, but can be completed, particularly with helping children anticipate what happens during a scan [13]. Adding any additional procedures to an MRI study that requires participation or wakefulness of a child has unknown tolerability.

Pediatric and young adult studies conducted with previous versions of a sealed CO_2_ titration system for purposes of CVR measurements report successful scanning with the device in ages 9 years to 88 years [6–8]. As studies to date have had only a few pediatric patients, it is unknown how easily this methodology could be scaled to a larger pediatric population, including those with possible chronic diseases, such as sickle cell disease or asthma. Known potential symptoms of hypercapnia include headaches, shortness of breath, and lightheadedness with risk of panic and claustrophobia [8, 14]. For a child, these side-effects can exacerbate the discomforts already posed by the MRI scan alone. We hypothesized that with anticipatory guidance, children will tolerate MRI with inhaled CO_2_ through a mask as well as children undergoing only MRI.

## Materials and methods

Children were enrolled in a prospective, single-site observational study, approved by the Institutional Review Board at the corresponding author’s institution from 2020 to 2024. Informed consent was obtained from each participant, or legal guardian if less than 18 years old upon enrollment. This data was collected alongside MRI data of two cerebrovascular imaging study groups at the same site, both of which recruited children with sickle cell disease and utilized BOLD imaging. Children who were recruited were without developmental delay or vasculopathy, as these were exclusion criteria. Participants were included if they were younger than 21 years old, attempted a study scan, and completed a post-scan questionnaire. If a participant had more than one study MRI, only the first MRI was included. Medications prescribed or taken regularly were reported at the time of the scan. A child was considered to have reactive airway disease if they reported daily or as needed inhaler use. Other variables collected included sex, age, height, weight, maximum end-tidal CO_2_, and baseline CO_2_.

**Fig. 1:**
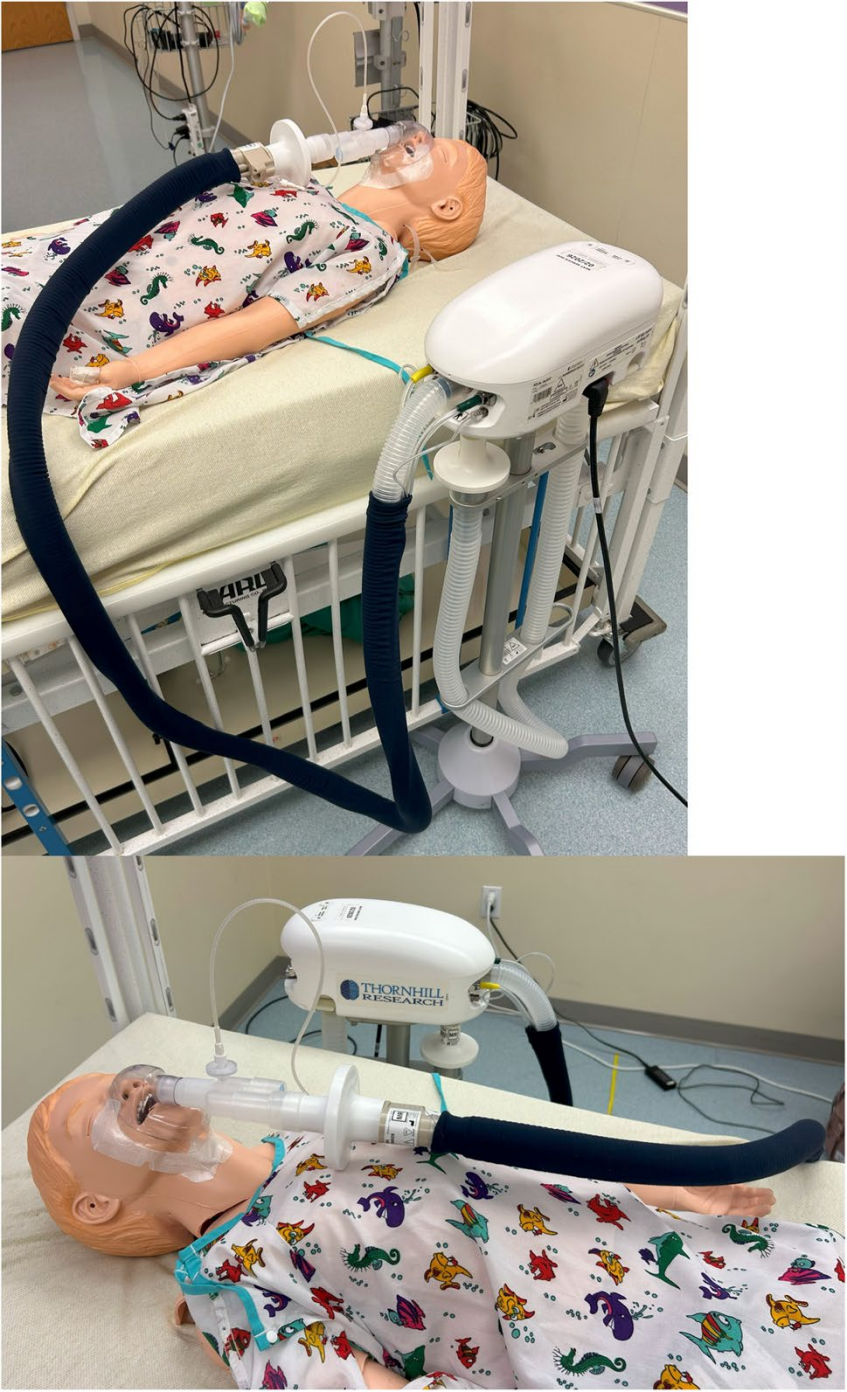
Mannequin demonstrating how the RespirAct® mask fits on a child’s face

A non-contrast brain MRI was performed via Siemens 3 T Prisma in all participants without sedation. All children changed into fabric or paper scrubs for the scan and were told briefly what to expect during the MRI scan. The participants undergoing MRI-measured CVR had concurrent end-tidal CO_2_ and oxygen control with recording using the RespirAct® device via a sealed mask. The RespirAct® (Thornhill Research Inc. Toronto, Canada, Gen 4) is a computer-controlled gas mixer that clamps participants’ individual end-tidal CO_2_ and titrates CO_2_ levels accordingly to measure CVR (Fig. 1) [15]. The study team spent approximately 10 min providing coaching language regarding the CVR procedure to each participant during consenting and during the masking process (Table 1). This explanation included telling CVR participants that they may or may not notice a change in their breathing pattern when the CO_2_ levels changed. For younger participants, the study team sometimes described the MRI and CVR portion of the study as an astronaut going into outer space with the scanner as the spaceship and the RespirAct® mask as a space suit breathing apparatus. Children in the CVR study were given the opportunity to decorate their masks using markers, decorative tape, and other themed stickers if desired. The disposable mask was cut to ensure it would cover the participant’s nose and mouth and create a more comfortable, custom fit for the participant. The mask was secured to the participant’s face using a transparent adhesive medical dressing. To ensure there was a secure seal around the mask, the participant was asked to breathe out while covering the openings of the mask. If a leak in the mask was identified, additional medical dressing was used to secure the mask. The masking process lasted for approximately 10 min. All participants wore ear protection and watched their choice of movie during the scan, except during BOLD with hypercapnic challenge, during which they viewed a projected white “ + ” symbol on a black background and were instructed to stay awake. During hypercapnic challenge, end-tidal CO_2_ levels were targeted to increase up to 10 mmHg above baseline end-tidal CO_2_. Specifically, we targeted acquiring 60 s of BOLD at the clamped baseline end-tidal CO_2_, followed by a “step” up 8 mmHg above the clamped baseline held for 60 s, a return to clamped baseline for 45 s, then a slow “ramp” over 150 s to 10 mmHg above baseline end-tidal CO_2_. The exact percentage of delivered CO_2_ was titrated by RespirAct® to target these levels. The remainder of the BOLD sequence was acquired targeting the clamped baseline value. The measured baseline and maximum capture end-tidal CO_2_ were recorded. CVR imaging was performed as the 2nd MRI sequence in the protocol, as anatomic MR sequences were obtained while establishing baseline and calibrating RespirAct® to the participant’s specific physiology. The mask remained in place and delivered a small amount of gas to maintain a steady-state infusion “clamped” baseline CO_2_ 1–3 mmHg above the participants’ naturally exhaled baseline throughout the duration of the scan, including post-CVR anatomical imaging. As the scans were for research purposes only, participants were informed that they could stop the scan if they were too uncomfortable or wanted to get out. The study team checked in on the participant throughout the scan. MRI images were evaluated during acquisition and non-CVR scans could be repeated if there were concerns about image data quality and the participant agreed to stay in the scanner to continue image acquisition. DICOM header timestamps were utilized to calculate total scan durations. Specifically, we extracted the Acquisition Time (DICOM Tag (0008,0032) from the first and last image of each study and included the full duration of the final sequence to precisely quantify the MRI session length. We report the total root‐mean‐square (RMS) movement at a 50-mm radius metric, which summarizes head displacement by computing the square root of the mean of squared motion parameters over all BOLD frames. We used 4dfp (four-dimensional functional processing) suite developed at our institution for automated fMRI preprocessing and analysis. Motion estimation is performed via the FALN (fast automated linear normalization) algorithm for intensity‐based alignment followed by XR3D (extended rigid-body cross-registration in 3D), which outputs framewise translations (in millimeters) and rotations (in degrees). To express total head motion on the cortical surface, 4dfp converts rotational root‐mean‐square into millimeters at a 50-mm radius and then combines it with translational root‐mean‐square.

**Table 1:** Sample coaching language. Examples of language used by study team when preparing young participants for MRI scan with RespirAct®

	Sample coaching language
During consenting, pre-MRI	• “The participant wears a mask while in the MRI. For part of the MRI, the carbon dioxide in the air you breathe will change slightly. This helps us capture images of the brain’s response to this.” • “Some people say it feels like they just jogged up a hill or ran up a flight of stairs while their air is being changed.” • “It’s important that our participants know what to expect for this MRI so they aren’t surprised that their breathing may change during the scan and know that it is normal to feel that change.” • *After describing a regular MRI*: “This MRI would be different because this would also involve a RespirAct® machine slightly changing the carbon dioxide that you breathe in. Carbon dioxide is a normal thing we breathe in and out from the air.” • “Your air doesn’t change for the whole scan, but when it does, it might feel like your body got tricked into thinking you’ve had a light workout. That can feel weird when you’re lying in an MRI. We can’t get good images of your brain if you ran up a hill and had a light workout outside, but we can get good images if your body changes as if you ran up a hill while you’re in the MRI.” • “Some people don’t notice when their air is being changed, but some people do. Some say they notice a smell; like if they opened a can of soda and could smell the bubbles. If you do notice the change, you just breathe normally and stay still for the MRI.”
During masking/MRI prep	• *While showing the mask*: “This part of the mask would be custom fit to your face and would be secured with a medical grade tape. The tape helps us get a good seal and holds the mask in place. These two spots are where hoses connect to the mask. When we connect those hoses, the RespirAct® machine will start telling me the make-up of the gases that you breathe normally so it knows what amount to safely change your air to.” • “I’m going to hold the mask up to your face so I can gage how we need to cut it to custom fit it for you. We want it to fit well so that you’ll be more comfortable with it on.” • “Do you feel like a scuba diver? Or like an elephant seal who grew a trunk?” • “Remember, your job is to just try to breathe normally, stay still, and squeeze this ball if you need us.”

**Fig. 2:**
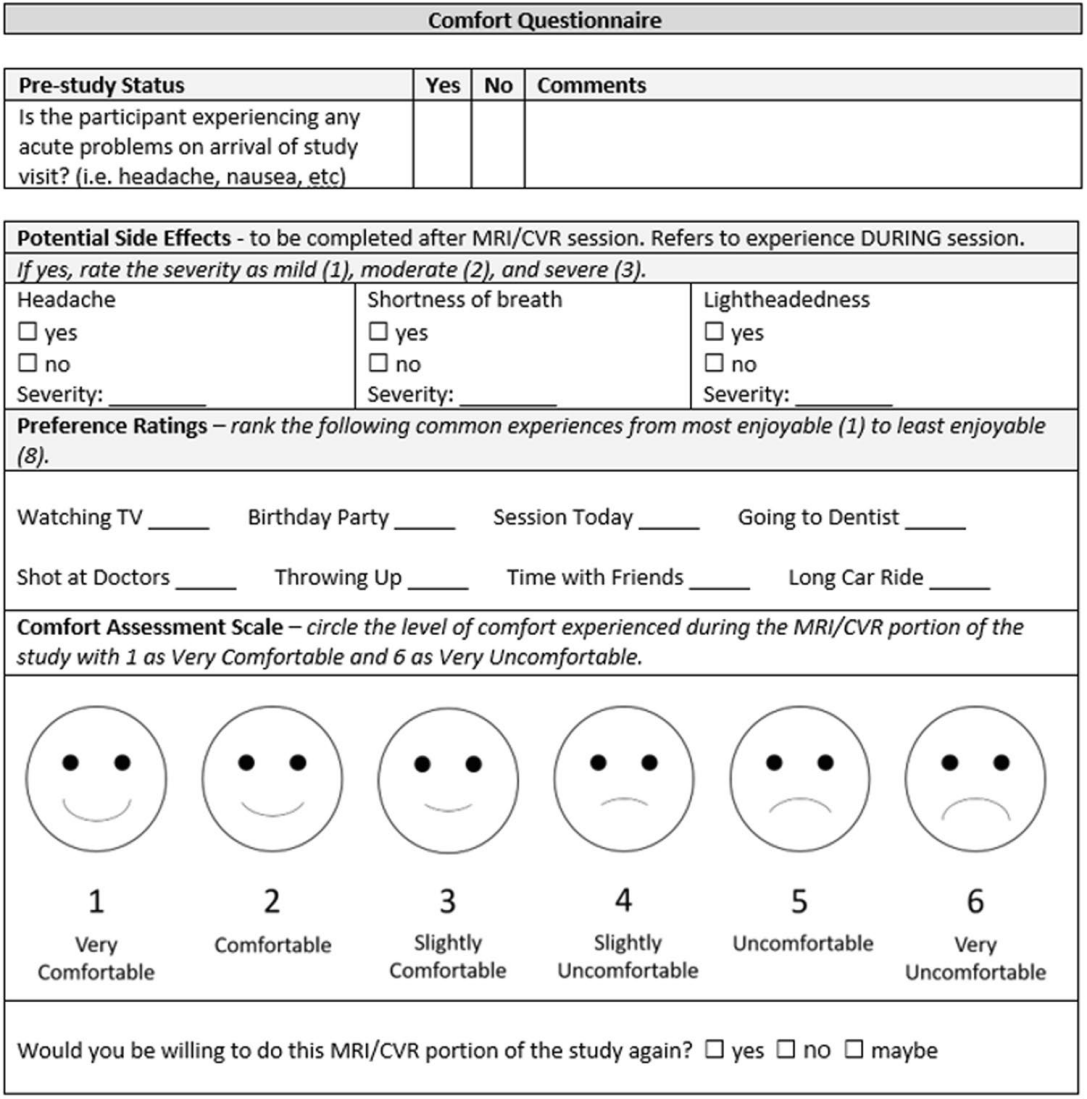
Post-scan questionnaire. CVR, cerebrovascular reactivity

Participants completed a post-scan questionnaire that asked about potential symptoms during and after the scan session with a severity ranking. Participants also ranked the scan in a preferential list of common childhood activities [16], completed a comfort scale, and reported whether they would be willing to return for the CVR or MRI only session of the study again with options of “Yes,” “No,” and “Maybe” (Fig. 2). Our post-scan questionnaire was modeled after participant questionnaires used in tolerability studies in non-invasive brain stimulation [16, 17]. The preferential ranking system asks participants to rank activities, one of which is the scan session they just completed, in order from most to least enjoyable (Fig. 2). This was chosen as it allows for similar levels of preference across a childhood age span and may give participants a familiar context in which to rate their experience. The comfort scale was designed for this study with a scale of 1 to 6 with accompanying faces, inspired by similar pediatric scales used to assess physical pain [18–20]. If any symptoms were reported, the study team assessed the participant at the end of the study visit and, if persisting, daily until resolved. In this study, tolerability was defined as being able to complete the MRI sequences required for the study without any lasting symptoms and without serious adverse events.

Statistical analysis was completed via Statistical Analysis System (SAS). Baseline continuous variables are presented as median and interquartile range (IQR) and categorical variables are presented as percentages. Comparisons of cohorts (MRI only and CVR imaging) were made with the Mann–Whitney *U* test for continuous variables. We performed univariate analysis between headache severity and maximum end-tidal CO_2_ with the Spearman rank correlation coefficient. We performed a multiple linear regression model to examine the relationship among time in MRI scanner and various measures of tolerability, including willingness to repeat the study, symptom severity (headache, shortness of breath, and lightheadedness), and comfort ratings to examine whether either children with less comfort may have prolonged scanning time (possibly from more time talking to team or making adjustments during the scan or may have needed to repeat images) or a shortened scanning time if the participant or team decided to forego optional or repeat imaging.

## Results

**Fig. 3:**
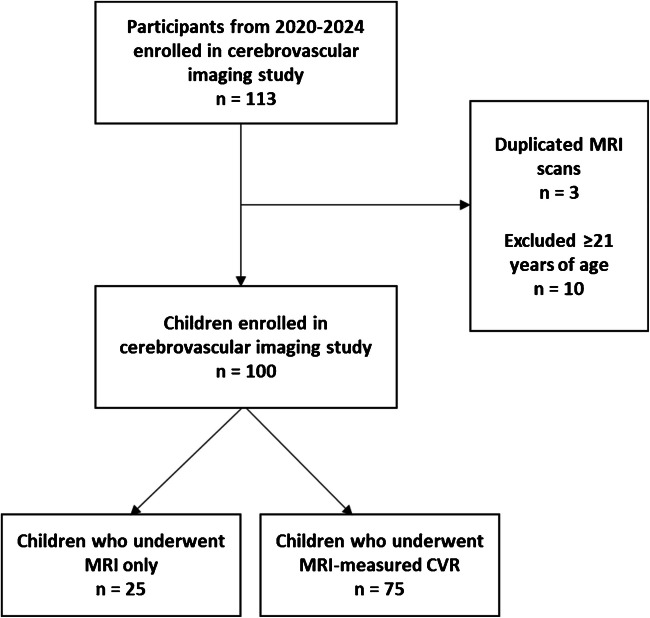
Flow diagram. CVR, cerebrovascular reactivity

**Fig. 4:**
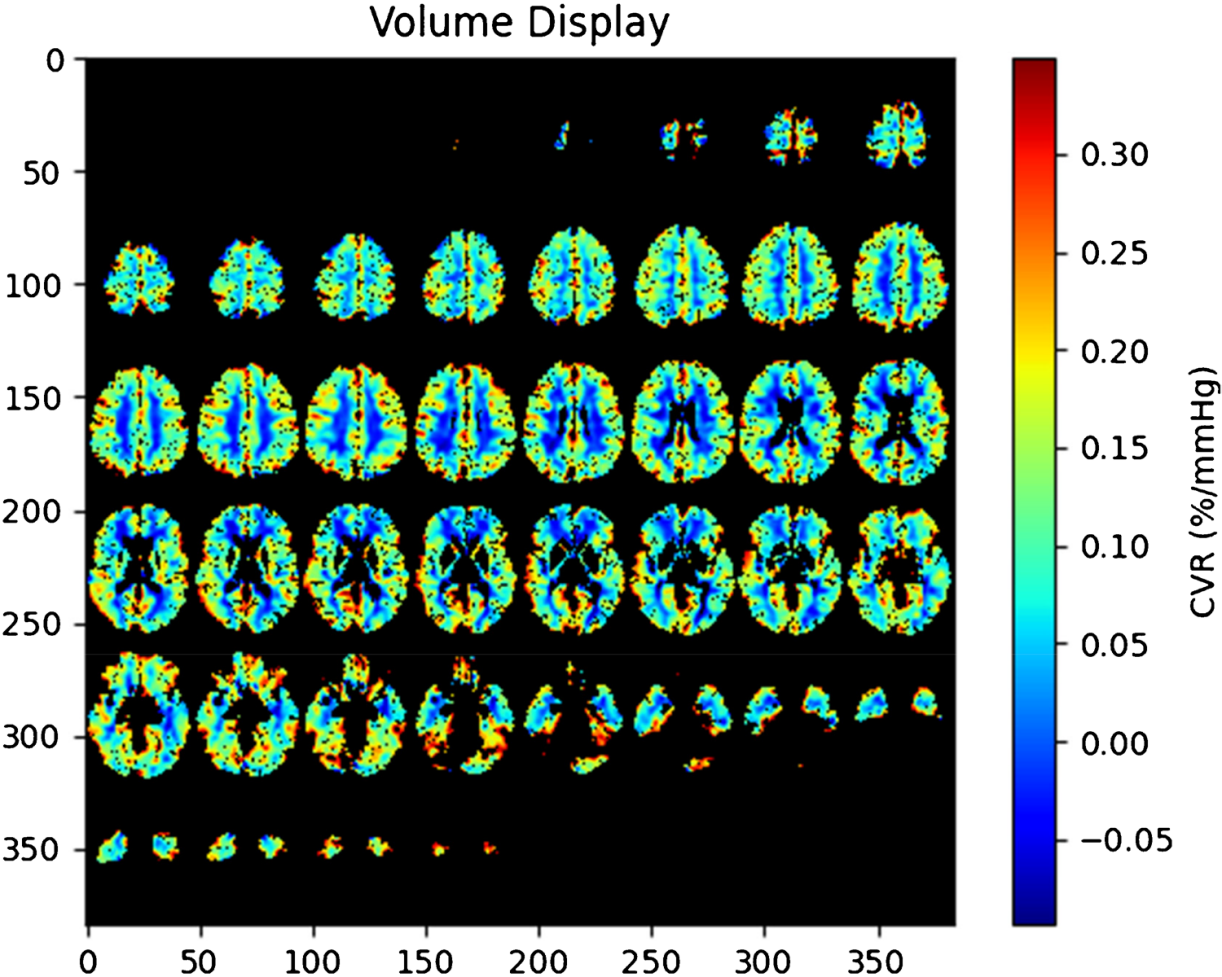
An example CVR map of an 8-year-old participant

A total of 113 MRI scan sessions were performed and had post-scan data available. Thirteen sessions were excluded as three had more than one scan session, and 10 sessions occurred in participants 21 years or older. The final cohort included 100 children, 75 (75%) of whom underwent MRI-measured CVR with RespirAct® (Fig. 3). Median age of all participants was 14 years, ranging from 5–20 years, with the CVR cohort ranging from 6–20 years. Across the entire population, 35 children had sickle cell disease (35%), and 16 had reactive airway disease (16%) (Table 2). Children in the MRI only cohort were more likely to have sickle cell disease and more likely to have had a prior MRI. Median duration of the scan time was 73.6 min for MRI with CVR, and 60.7 min for MRI only (*P*<0.0001). Children with CVR had a median clamped baseline end-tidal CO_2_ of 41.0 mmHg (39.0, 43.0) and experienced a median maximum end-tidal CO_2_ of 50.7 [47.9, 53.1]. There was not a difference in the quantified motion of BOLD scans between those done with and without hypercapnia (*P*=0.33, Table 2). Of the 75 children who underwent CVR imaging, 58 (77%) had quantifiable data. Those in whom CVR data was not able to meet quality control for processing had a higher root-mean-square of motion (2.62 [1.58, 4.73]), compared to those who had processable data (0.63 [0.37, 0.97], *P*<0.001. Children with CVR failure tended to be younger (median age of 11.42 vs 15.05, *P*<0.001), but CVR data was successfully acquired in children as young as 8 years old (Fig. 4). Two children who failed CVR imaging also had inadequate standard MR imaging. There was no relationship between degree of motion and either clamped baseline end-tidal CO_2_ (*P*=0.73) or maximum end-tidal CO_2_ level (*P*=0.99). Standard MR imaging did not reveal any significant abnormalities.

**Table 2:** Participant characteristics. Variables presented with mean and interquartile range or percentage of cohort with comparison of participants who underwent CVR session and those who underwent MRI only sessions. *ETCO*_*2*_, end-tidal carbon dioxide; *CVR*, cerebrovascular reactivity

Variables	All participants (*N*=80)	Participants with CVR session (*N*=55)	Participants with MRI only (*N*=25)	*P*-value
Age	14.0 [11.0, 16.3]	14.4 [11.4, 17.4]	13.0 [10.0, 15.0]	0.07
Sex, female	59/100 (59.0%)	43/75 (57.3%)	16/25 (64.0%)	0.56
Sickle cell disease	35/100 (35.0%)	20/75 (26.7%)	15/25 (60.0%)	**0.003**
Prior MRI	63/100 (63.0%)	43/75 (57.3%)	20/25 (80.0%)	**0.04**
Reactive airway disease	16/100 (16.0%)	11/75 (14.7%)	5/25 (20.0%)	0.53
Height (cm)	157.5 [146.1, 167.6]	161.8 [149.9, 167.6]	152.9 [135.9,162.8]	**0.009**
Weight (kg)	54.4 [40.4, 66.6]	55.0 [41.8, 67.1]	48.6 [36.6, 64.1]	0.24
MRI duration (minutes)	71.9 [63.6, 77.7]	73.6 [68.8, 81.9]	60.7 [54.1, 64.8]	**<0.0001**
Root mean square of frame motion	0.71 [0.45, 1.58]	0.68 [0.41, 1.37]	0.82 [0.54, 1.73]	0.33
Baseline ETCO_2_ (mmHg)		41.7 [39.9, 43.0]	N/A	na
Maximum ETCO_2_ (mmHg)		52.7 [49.8, 55.5]	N/A	na

Although headache, shortness of breath, and lightheadedness were all reported as symptoms experienced during the scan in both groups, a child was more likely to endorse symptoms with a CVR session than with MRI only, the most common being shortness of breath (42/75 CVR sessions, *P*<0.001) (Table 3). We compared univariate analyses of maximum end-tidal CO_2_ and headache severity (mild and moderate) and did not find a relationship (*P*=0.28). There was a correlation between age, however, as younger children were more likely to report higher headache severity scores (*P*=0.006) whereas older age was associated with reporting shortness of breath (*P*=0.04). There was not a correlation between age and lightheadedness reporting. Eleven children in the CVR cohort reported a mild symptom after the MRI at the end of the study, either headache or lightheadedness, which was an improvement from symptoms reported during the CVR session. No serious adverse event occurred, and all symptoms eventually resolved without intervention. Within the CVR group, we evaluated the effect of having reactive airway disease on symptom reporting. There was no difference in symptom reporting in participants with or without reactive airway disease (*P*=0.70).

**Table 3:** Symptoms participants reported experiencing during MRI with cerebrovascular reactivity (CVR) testing vs MRI only

Symptom during scan	Severity	CVR session (*N*=55)	MRI only (*N*=25)	*P*-value
Headache		34	2	**0.0008**
	Mild	22	1	**0.009**
	Moderate	12	1	0.12
	Severe	0	0	—
Shortness of breath		42	3	**0.0001**
	Mild	24	3	0.05
	Moderate	17	0	—
	Severe	1	0	—
Lightheadedness		19	2	0.07
	Mild	15	1	0.06
	Moderate	4	1	—
	Severe	0	0	—

**Fig. 5:**
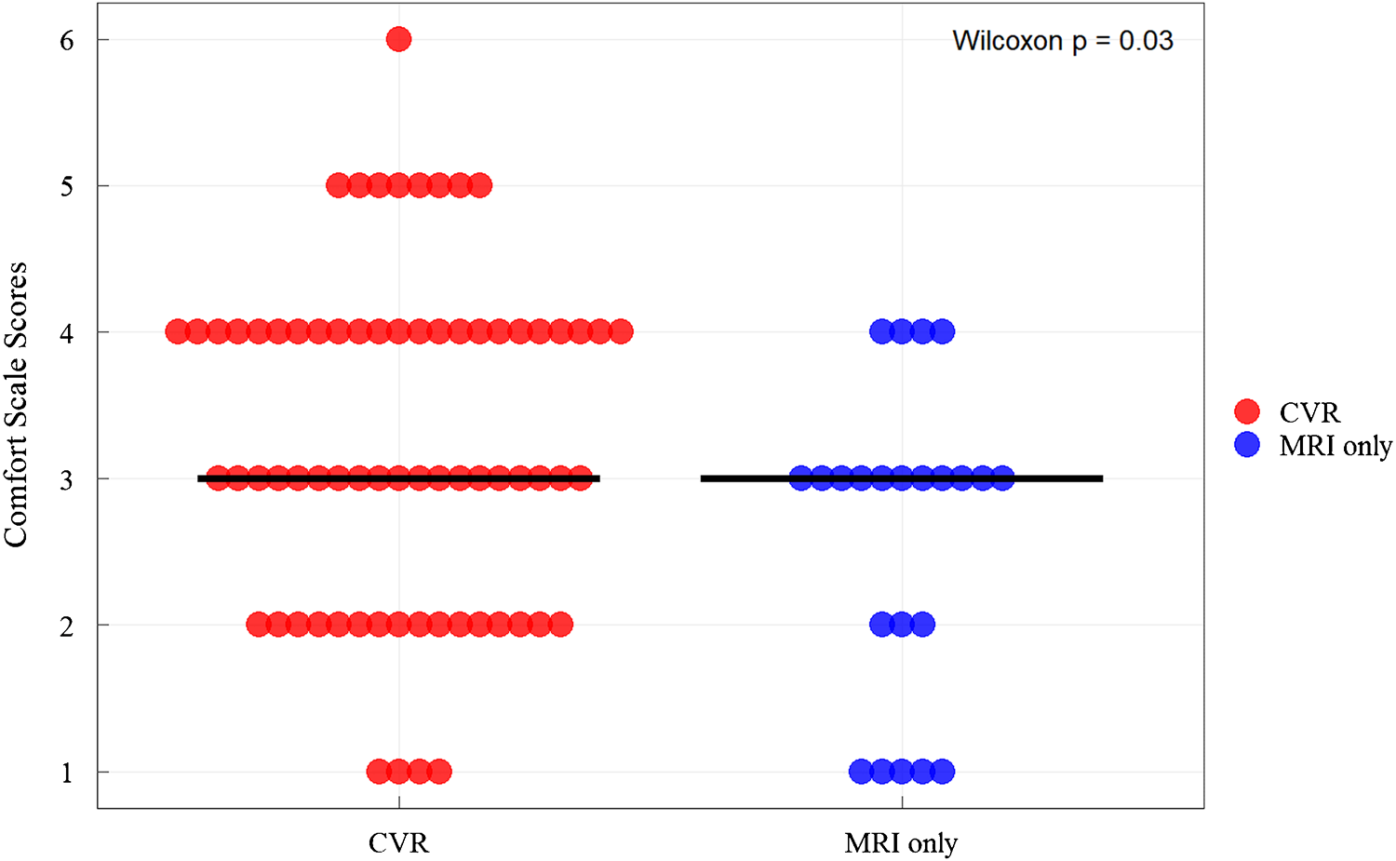
Comfort scale scores. Although the cerebrovascular reactivity (CVR) cohort and MRI only cohort both had a median comfort score of 3, the CVR cohort had a wider distribution of scores reported. On this scale, 1 was “very comfortable” and 6 was “very uncomfortable.” Each dot represents a participant’s rating. Red dots are participants who underwent MRI with CVR and blue dots represent participants whose scan did not have CVR attempted

Even though the median comfort scale was the same (3 = slightly comfortable), in both CVR (3 [2, 4]) and MRI-only (3 [2, 3]) cohorts, the Mann–Whitney *U* test revealed a significant difference in the rank of comfort ratings between the two groups (*P*=0.03; Fig. 5). Specifically, a higher proportion of children reported lower comfort ratings (4–6) in the CVR cohort (32/71) compared to the MRI-only cohort (4/23).

**Fig. 6:**
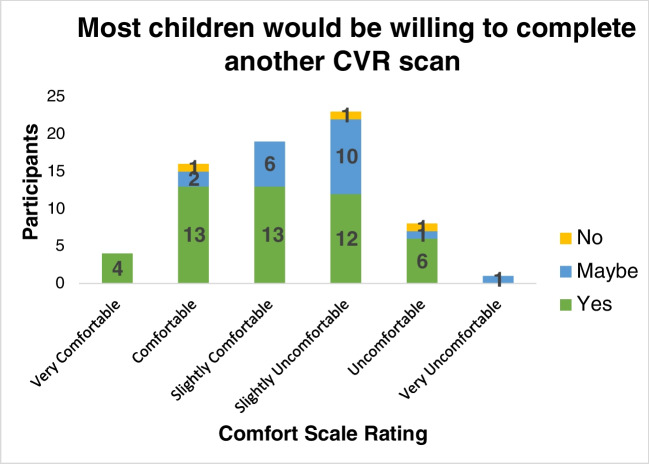
Willingness to repeat CVR scan, based on comfort scale reported. Even children who reported the scan as being uncomfortable endorse a willingness to repeat a similar scan in the future

The majority of children undergoing a CVR scan noted that they would definitely be willing to do the CVR session again (51/74, 69%), and an additional 20 reported that they may be willing to do a CVR scan again, even among those who noted discomfort (Fig. 6). One participant had a missing response. The ages of the children unwilling to repeat the scan were 6 years, 11 years, and 12 years. There was no difference in comfort reporting of a CVR session between children with and without sickle cell disease (*P*=0.54).

**Fig. 7:**
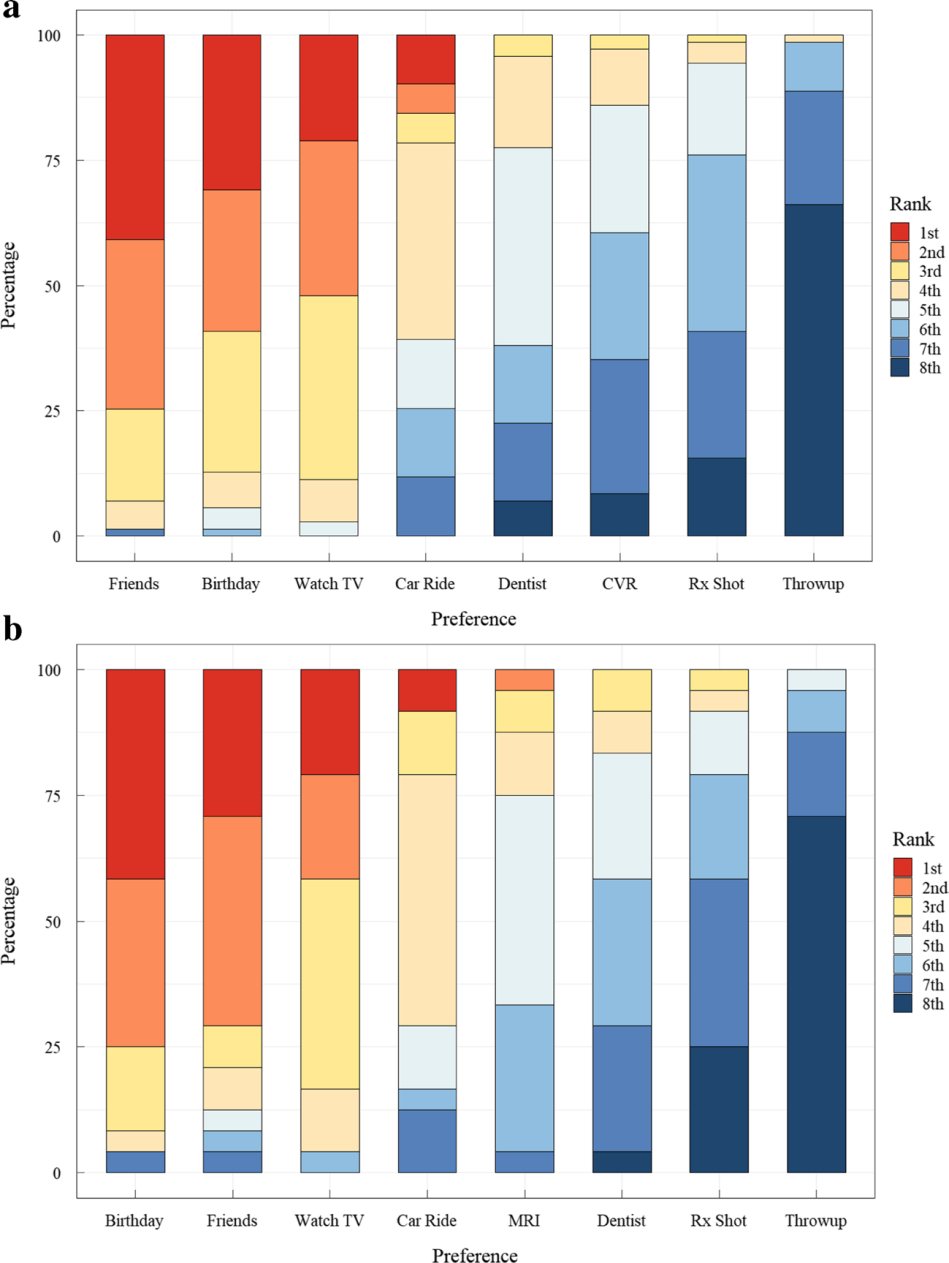
Preference rankings among cerebrovascular reactivity (CVR) cohort children rated each activity in order of most enjoyable (1st) to least enjoyable (8th), the frequency distribution of each activity rated among children who had CVR MRI is shown in panel (**A**) (*n*=71), compared to standard MRI only in panel (**B**) (*n*=24)

When evaluating preference ratings of eight activities, children in our cohort did not have differences in their rankings, apart from where CVR and/or MRI ranked. Five children had incomplete rankings and were excluded from preference analysis. The majority of children rated “Time with Friends” as 1 (36/95, 38%), followed by “Birthday Party” as the second most common top-rated choice 34% (32/95). Children who underwent CVR session ranked their scan experience most commonly as 7th (19/71, 27%) as compared to 5th placement with MRI only (10/24, 42%). The CVR session was ranked similarly to going to the dentist and getting a shot at the doctor’s office. Only 6 children ranked the CVR session as the least preferred activity. Throwing up was most commonly ranked as the least preferred activity (Fig. 7).

In a multivariable linear regression, headache, shortness of breath, lightheadedness, willingness to repeat scan, or comfort rating, were not significantly associated with MRI scan duration (all variables *P*>0.36). Furthermore, the model has very limited explanatory power, with an *R*-squared value of 0.02, indicating that the included variables explain little to no variation in scan duration. These results suggest that MRI scan duration is not significantly influenced by any of the tolerability measures assessed in this study, and participants’ subjective experiences, such as discomfort or symptom severity, have minimal impact on the actual procedure.

## Discussion

This prospective study shows that unsedated advanced neuroimaging incorporating measurement of CVR by administering exogenous CO_2_ was overall well tolerated in children as young as 6 years of age, although the youngest with usable data was 8 years of age. All children were able to complete the MRI session. As compared to the MRI only group, children undergoing measured CVR did report more symptoms, although none were serious or long-lasting. Interestingly, a few children undergoing only MRI also reported symptoms of headache, shortness of breath or lightheadedness (*n*=6/25). Children with chronic disease, including sickle cell disease and reactive airway disease, did not have a difference in reported comfort during the scan. This finding is important for both clinical and research investigations as it gives evidence that changing CO_2_ for brain imaging is not contraindicated in developmentally normal children and can be completed safely in common childhood conditions such as reactive airway disease. However, this study did not include children with known intracranial disease so does not address all childhood conditions. In our study, MRI scan duration did not contribute to the tolerability of the procedure itself, with all scans being close to an hour.

Our results are congruent with symptoms of similar imaging methodology in a cohort of 294 healthy individuals across the lifespan, which included 59 participants ages 9–19 years, reported by Spano et al. [8]. Symptoms were not specified by age range in this study, but minimal symptoms were reported across the cohort, with the most common being shortness of breath (6.5%), followed by anxiety/claustrophobia (2.5%), headache, and lightheadedness (0.5% each). The type of symptoms experienced by our participants was similar, with shortness of breath being most common (56%), followed by headache and lightheadedness. We did not specifically ask about claustrophobia in our study, but this is unlikely as the participants completed the hour-long MRI scans. The increased incidence of symptoms from our cohort is likely explained not only by the younger age range but also by the prospective nature of our study. Due to the retrospective nature of their study, Spano et al. were only able to detect symptoms that were significant enough to be documented in the research records.

Of our CVR session participants prospectively asked about their comfort level on a scale of 1–6, 45% (32/71) reported experiencing some discomfort (score 4–6). This is higher than the 11.1% in Spano et al. retrospective cohort who had noted discomfort [8]. However, in our cohort, only 9 (13%) selected the uncomfortable or very uncomfortable response, which may be a more accurate comparison. Spano et al. noted that half of those who had reported discomfort had a repeat scan in the database, demonstrating that the discomfort was not severe enough to discourage further participation. Similarly, the majority of our participants who found the scan uncomfortable reported that they would be willing to complete the scan again. Importantly, there were no serious adverse events in either study, supporting the safety of this methodology.

The preference scale has advantages for translating the research experience to common childhood activities and has been used in prior studies [16, 17]. It allows for pediatric age-appropriate ranking of any intervention within a grouping of other activities that are universally unliked (vomiting) and enjoyable (spending time with friends). Thus, investigators can easily compare rankings across a cohort while comparisons are made between groups to evaluate similarities of the population in interests and the intervention or methodology being studied. However, it also has limitations. First, while it lists common experiences, some have a bias based on socioeconomic status. Some children who participated in the study live in an urban setting and required transportation assistance to the scan. They may have different experiences and perception of “long” car rides, possibly not having taken many road trips longer than an hour. Other children may have taken multi-hour road trips, but with the ability to watch movies on devices. Modern handheld devices were not available when the scale was created in the late 1990s. Even with these limitations, it still communicates that the MRI, with or without CVR measurement, was not the most enjoyable activity (as expected) but also that most children did not consider it the worst option on the list.

Importantly, only 3 children undergoing MRI measured CVR reported that they would not be willing to repeat the scan in the future, and 51 would definitely be willing to repeat the scan visit. The option of “maybe” was included to ensure that children who were unsure of repeating the scan or of possible consequences of their response had an option other than “no” to report and thus strengthen our confidence in the sincerity of the “yes” response. This provides context for both the level of discomfort and the ranking of the scan, and it is encouraging data for the feasibility of longitudinal studies.

There are several limitations of this study. First, we include a potentially biased research sample of those who consented to participate in an MRI research study. It is unknown how children who declined to participate may or may not tolerate the imaging without sedation. Second, while children with chronic conditions were included, they were otherwise healthy and feeling well at the time of the research scan. However, this is likely similar to clinical imaging, with the exception of urgent imaging needs. Third, this is a developmentally appropriate cohort. These findings cannot be extrapolated to children who are not at the cognitive level of an elementary school child, either by developmental stage or cognitive impairment. Fourth, we did not include children with known intracranial disease, so it is unknown whether or not that intracranial disease would impact these findings. Fifth,we did not use the common terminology criteria for adverse event (CTCAE) [21]. By this scale, ranging from mild to causing death, all symptoms reported would be categorized as mild, as none required interventions. This would have impeded our primary interest in capturing more subtle variations of children’s perception of their experience, including symptoms that spontaneously resolved. An additional limitation is that while it is one of the largest pediatric cohorts of exogenous CO_2_ imaging reported to date, it is still a relatively small sample size, and a larger cohort may discover more symptoms or difficulty tolerating the scan.

## Conclusion

Children as young as 8 years old, including children with sickle cell disease or reactive airway disease, can achieve unsedated advanced imaging with exogenous carbon dioxide.

This manuscript is the result of funding in whole or in part by the National Institutes of Health (NIH). It is subject to the NIH Public Access Policy. Through acceptance of this federal funding, NIH has been given a right to make this manuscript publicly available in PubMed Central upon the Official Date of Publication, as defined by NIH.

## Data Availability

Data availability statement: The data supporting the findings of this study are available from the corresponding author upon reasonable request.
